# Spatiotemporal evolution and ecological pathways of riparian forests within the ecosystem service cascade framework: a comprehensive analysis from the Irtysh River Basin, China

**DOI:** 10.3389/fpls.2026.1904769

**Published:** 2026-08-19

**Authors:** Ye Yuan, Guangming Chu, Zongchi Fu, Tong Liu, Wenli Wu

**Affiliations:** 1 College of Life Science, Shihezi University, Shihezi, China; 2 Xinjiang Production and Construction Corps Key Laboratory of Oasis Town and Mountain-basin System Ecology, Shihezi, China; 3 Country College of Urban and Environmental Sciences, Shihezi University, Shihezi, China

**Keywords:** ecosystem services, Irtysh River Basin, land use change, riparian forest, vegetation dynamic

## Abstract

**Introduction:**

Riparian forests in the valley area of the Irtysh River Basin (IRB) play a vital role in regulating regional hydrological and carbon processes, and they also serve as critical transboundary habitats for endangered species. However, these forests have been increasingly affected by human activities such as agricultural cultivation and livestock grazing.

**Methods:**

This study examines the spatial distribution and temporal trends of land use and cover change, and vegetation greenness in the riparian forests in the valley area of the IRB from 1990 to 2020, and assessed four key ecosystem services (water retention, soil conservation, habitat quality, and carbon storage) with the InVEST model. In addition, the influences of land use and cover patterns, vegetation greenness, and other socio-ecological factors on integrated ecosystem services were quantified.

**Results:**

The results showed that: (1) over the past 30 years, forest cover area declined substantially. Along the main stream of the Irtysh River, which has the largest forested area, the forested area has declined from 352.71 km^2^ to 163.10 km^2^. Forest land was primarily converted into sparse woodland and grassland; (2) high vegetation greenness grade exhibited a clear spatial pattern extending along both sides of the river channel. The multi-year mean maximum NDVI remained relatively high at 0.50, with an increasing trend; (3) except for soil conservation, the other three ecosystem services showed strong spatial heterogeneity. From 1990 to 2020, water retention and habitat quality remained relatively stable, whereas soil conservation and carbon storage both declined; (4) land use intensity and vegetation greenness exerted significant negative and positive effects on integrated ecosystem services, with path coefficients of −0.38 and 0.27, respectively.

**Discussion:**

The findings reveal in clear detail the long-term dynamics and ecological functions of riparian forests in the valley area of the IRB, thereby offering important insights for their future conservation and restoration.

## 1 Introduction

Ecosystem service assessment is not only a quantitative approach to evaluating natural capital but also an essential pathway for achieving regional sustainable management (Albert et al., 2016; Yu and Hao, 2020). In 2001, the Millennium Ecosystem Assessment (MA), initiated by UN Secretary-General Kofi Annan, defined ecosystem services (ES) as the direct or indirect benefits humans obtain from ecosystems (Fisher et al., 2009; Millennium Ecosystem Assesment, 2005), and classified them into four categories: provisioning, regulating, cultural, and supporting services (Zhao and Zhang, 2006). Over the past two decades, ecosystem service research has evolved from the quantification of individual ecosystem services toward increasingly integrated assessments of ecosystem functioning and sustainability. As research has progressed, increasing attention has been shifted from the quantitative assessment of ecosystem services to the analysis of their formation mechanisms. Ensuring the sustained supply of ecosystem services has since become a central research focus, encompassing ecosystem service supply–demand balance (Chen et al., 2022; Lei et al., 2024), trade-offs and synergies (Wang et al., 2019), and the drivers of ecosystem service change (Pan et al., 2020). In recent years, scholars worldwide have increasingly adopted interdisciplinary frameworks that integrate ecological and socio-economic characteristics to address global environmental challenges such as biodiversity loss (Fu et al., 2022; Shrivastava et al., 2020), contributing to the emerging “pattern–process–service–sustainability” research paradigm (Fu et al., 2025). At the same time, the complex interactions between human activities and ecosystem services highlight the need to shift away from traditional top-down ecosystem management (Chen and Liang, 2023; Chen L, et al., 2025).

Approximately 38% of global terrestrial borders traverse climate-sensitive regions, biodiversity hotspots, or transboundary river basins (Wang et al., 2025). As essential components of azonal vegetation in arid landscapes (Zhang et al., 2026), riparian forests are among the most biologically productive ecosystems worldwide, despite occupying only a narrow transition between terrestrial and aquatic environments (Xu et al., 2022). Originating in the southern Altai Mountains, the Irtysh River flows 633 km within China before entering Kazakhstan and ultimately joining the Ob River in Russia (Huang et al., 2021). Influenced by valley topography and periodic flooding, riparian forests are widely distributed along major tributaries such as the Crane, Burqin, and Haba rivers. These forests represent the only natural habitats for certain rare species of *Salicaceae* (Qu et al., 2025). In addition to contributing to regional water–sediment regulation and carbon storage, the riparian forests of the IRB serve as crucial migration corridors and refuges for endangered species (Cao et al., 2015), and provide a multiple ecosystem services including forage resources, environmental education, and ecotourism, all of which benefit from the riparian forests and grasslands (Yu et al., 2025). Their ecosystem functioning is jointly regulated by natural factors such as river hydrology, flood disturbance, and climate variability, making ecological responses substantially more complex than those observed in many terrestrial ecosystems.

At the same time, land use change in riparian zones caused by human activities often triggers cascading ecological responses that extend beyond landscape transformation. Under a development model primarily reliant on the input of production factors (land, labor, etc.) (Fan et al., 2019; Jiang et al., 2019), the Altay region has faced a series of ecological and environmental challenges since the 1950s, including aging forest stands (Bai et al., 2004), grassland degradation caused by overgrazing (Liu et al., 2021b), and landscape fragmentation resulting from agricultural expansion (Jing et al., 2008). These pressures are particularly pronounced in arid and semi-arid regions, where riparian forests rely heavily on limited water resources and therefore exhibit high ecological sensitivity to both natural environmental variability and anthropogenic disturbances. Consequently, these characteristics make riparian forests in the valley area of the Irtysh River Basin ideal natural laboratories for investigating ecological pathways linking landscape change with ecosystem service dynamics.

According to the feedback mechanisms underlying ecosystem service dynamics (Bai et al., 2022), the level of ecosystem services depends on the structure and functioning of ecosystems. Land systems serve as the foundation for ecosystem services, and changes in land use types directly influence ecosystem components and spatial configurations (Fu and Zhang, 2014). The Normalized Difference Vegetation Index (NDVI) is a key indicator for measuring vegetation cover and growth status, and also effectively reflects ecosystem structure (Yang et al., 2019). In forest ecosystems, changes in forest area and vegetation greenness reveal historical trajectories of degradation or recovery. However, increases in forest area or NDVI do not necessarily imply that forest functions have recovered at the same rate or to the same extent (Mansourian, 2018). Therefore, long-term analyses of land-cover change and vegetation greenness are necessary to clarify their impacts on ecosystem services.

Previous studies have contributed significantly to ecological conservation and restoration in the IRB. For example, ecological quality has been assessed using improved eco-hydrological indices such as AWBEI (Luo et al., 2025b); the spatial pattern and temporal evolution of wetland landscapes have been used to quantify and predict carbon storage changes; and integrated evaluations of ecosystem services, ecosystem vulnerability (Luo et al., 2025a), and integrity have identified transboundary conservation priority areas in the Altai Mountains (Duan et al., 2024). Although some studies have evaluated the effectiveness of ecological restoration strategies based on ecosystem services (Liu et al., 2022, 2019). Unfortunately, they generally describe patterns of association rather than the ecological pathways through which environmental change influences ecosystem functioning, and two major limitations remain: (1) relatively few have explicitly integrated landscape transformation, vegetation dynamics, and ecosystem service provision within a unified ecological framework, and (2) most research has targeted the broader Altay region (Fu et al., 2016) or the entire IRB (Luo et al., 2022), rather than specifically focusing on riparian forest areas. Arid and semi-arid regions riparian forests remain substantially underrepresented in ecosystem service research.

Accordingly, this study integrates landscape transformation analysis, vegetation functioning assessment, ecosystem service evaluation, and ecological pathway analysis within the ecosystem service cascade framework. Such a process-oriented approach aims to: (1) analyze the spatiotemporal patterns of land use and cover change, and vegetation greenness within riparian forests in the valley area of the IRB; (2) quantify the spatial heterogeneity of four key ecosystem services (water retention, soil conservation, habitat quality, and carbon storage) from 1990 to 2020; and (3) evaluate the effects of factors such as land use intensity and vegetation greenness on ecosystem service enhancement. The findings are expected to systematically reveal the historical dynamics of riparian forests, a more comprehensive understanding of ecosystem service regulation, and developing sustainable management strategies for dryland riparian forests.

## 2 Materials and methods

### 2.1 Overview of the study area

#### 2.1.1 Study site

The Irtysh River Basin is located in the Altay region of northwestern China (85°31′36″–91°04′23″E, 45°00′00″–49°10′45″N), with a total watershed area of 52,730 km^2^. It represents a typical mountain–oasis–desert composite ecosystem in the arid and semi-arid zones of Central Asia. Within this system, water serves as the principal medium linking mountains, oases, and deserts (Li et al., 2018). The IRB supports three typical vegetation community types: taiga forest, riparian forest, and grassland. The region has a typical temperate continental climate, with a mean annual temperature of 2.3–6.9°C. Mean annual precipitation reaches approximately 500 mm in mountainous areas but only 150 mm on the plains (Qu et al., 2025).

#### 2.1.2 Division of forest zones in the IRB

**Figure 1:**
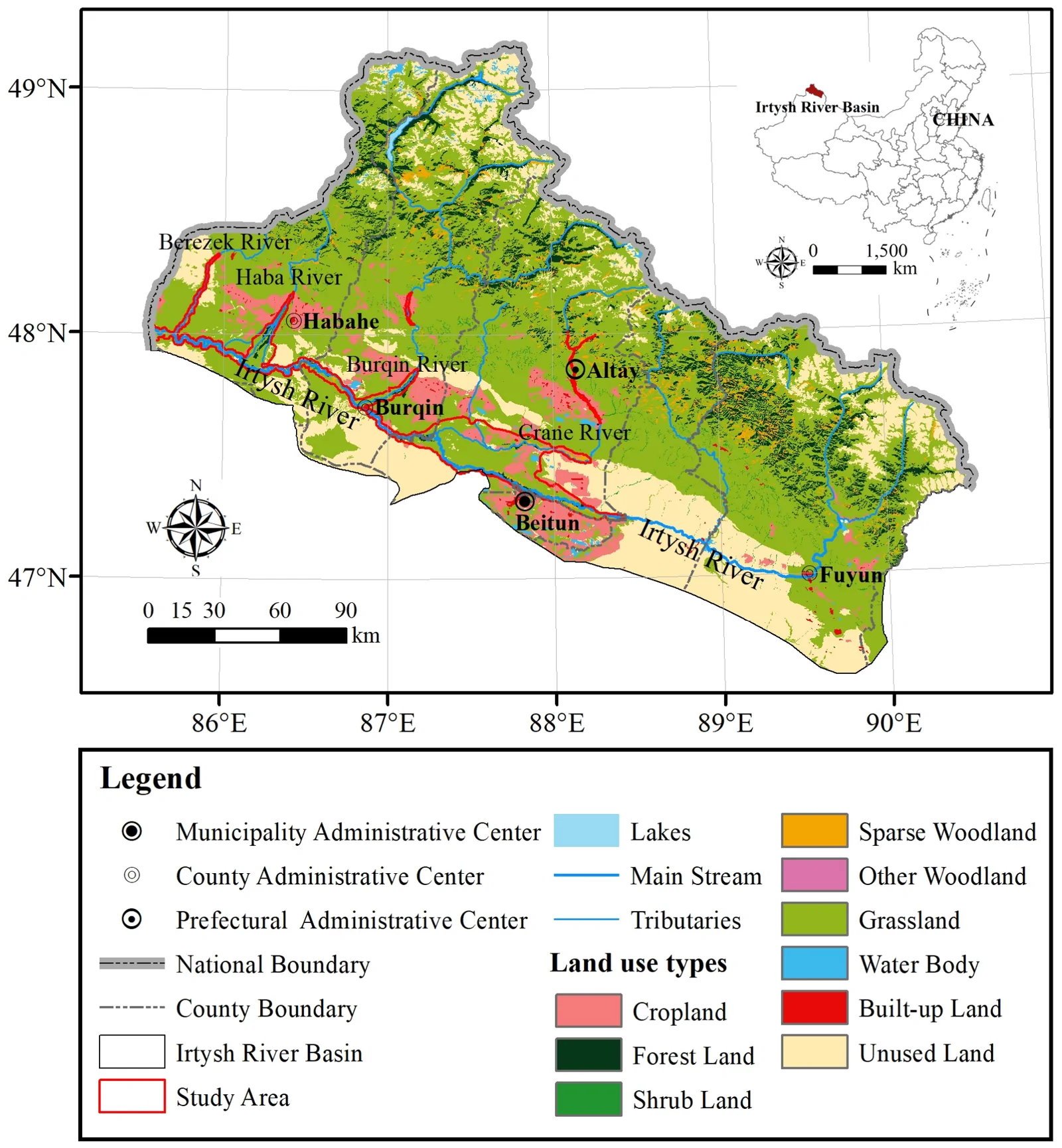
Map of the typical distribution area of riparian forests in the valley area of the IRB.

The study focused on representative riparian forest along the valley areas of the IRB (**Figure 1**). Based on the Vegetation Map of the People’s Republic of China (1:1,000,000) (Zhang, 2007), the temporal evolution of ecological flow processes (Deng, 2023), findings from previous studies on riparian forest protection and restoration (Cheng et al., 2006; Deng, 2024), and multi-year field surveys, the riparian forests of the IRB were classified into five major forest zones (**Supplemantary Appendix Figure B1**): (1) Irtysh River (IR) riparian forest zone (main stream), (2) Burqin River (BUR); (3) Crane River (CR); (4) Haba River (HR), and (5) Berezek River (BER) riparian forest zone (tributaries). These forest zones are represented by the corresponding river names in subsequent sections.

### 2.2 Data source and preparation

In this study, ArcGIS 10.8 was used as the GIS software platform. All raster and vector data (**Table 1**) were projected to the WGS1984–UTM Zone 45N coordinate system, with a uniform spatial resolution of 1 km × 1 km required by the InVEST model.

**Table 1:** Data source and description.

Data	Source link	Resolution
LUCC	Resource and Environmental Science Data Platform https://www.resdc.cn	30 m
NDVI	National Ecosystem Science Data Center https://nesdc.org.cn/	30 m
Precipitation	National Earth System Science Data Center http://www.geodata.cn	Monthly, 1 km×1 km
Evapotranspiration	National Earth System Science Data Center http://www.geodata.cn	Monthly, 1 km×1 km
Soil properties	National Cryosphere Desert Data Center https://www.crensed.ac.cn/portal/	1 km×1 km
DEM	Resource and Environmental Science Data Platform https://www.resdc.cn	1 km×1 km
GDP	Resource and Environmental Science Data Platform https://www.resdc.cn	1 km×1 km
Administrative divisions	National Catalogue Service For Geographic Information https://www.webmap.cn/	–

Land use and cover (LUCC) data were extracted from the China Multi-period Land Use Remote Sensing Monitoring Database, generated from visual interpretation of Landsat TM/ETM+/OLI imagery. Normalized Difference Vegetation Index (NDVI) data were obtained from the China Annual Maximum NDVI Dataset (30 m, 2000-2022). This dataset shows the annual maximum values of NDVI, representing the upper limit of optimal vegetation conditions. Precipitation (PRE) and rainfall erosivity factors were derived from the 1-km monthly precipitation dataset for China (1901-2024). Potential evapotranspiration (PET) data were extracted from the 1-km monthly potential evapotranspiration dataset for China (1901-2024). Soil parameters (maximum root depth, plant available water capacity, soil erodibility factor) were obtained from the Harmonized World Soil Database (HWSD v1.1). Elevation data was based on China’s DEM data at 1 km, 500 m, and 250 m resolutions (SRTM 90 m), which was resampled from the Shuttle Radar Topography Mission (SRTM V4.1). Gross Domestic Product data sourced from the Kilometer Grid Dataset of China’s GDP Spatial Distribution Dataset, this data was spatially interpolated based on county-level GDP statistics, nighttime lights, land use types, and settlement density. The basic geographic information data in the Altay Prefecture was provided by the National Catalogue Service for Geographic Information.

In addition to raster and vector data, tabular data such as biophysical tables, threat layers, sensitivity scores, and carbon density parameters were set according to the requirements of InVEST.

### 2.3 Quantification of land use and cover dynamics

Three typical years (1990, 2005, and 2020) were selected in this study. Based on the classification system of the dataset and research requirements, land use and cover types were aggregated into six primary categories: cropland, woodland, grassland, water body, built-up land, and unused land. To gain a deeper understanding of the variation characteristics of riparian forests, woodland was further subdivided into four secondary categories: forest land, shrub land, sparse woodland, and other woodland.

The land use transition matrix describes both the direction and magnitude of conversions between different land use categories. By summarizing the increases or decreases in various land use types at the beginning and end of the study period, it reflects the spatial and functional changes in land use patterns, as expressed in (Equation 1). The proportional chord diagrams illustrating the area proportions of land use transitions were generated in Origin 2022.

(1) $$S_{ij}=\left[\begin{array}{cccc} S_{11} & S_{12} & \ldots & S_{1n\;}\\ S_{21} & S_{22} & \ldots & S_{2n\;}\\ \ldots & \ldots & \ldots & \ldots \\ S_{n1} & S_{n2\;} & \ldots & S_{nn\;} \end{array}\right]$$

where *S_ij_* represents the area (km^2^) converted from land use type *i_th_* to type *j_th_*, and *n* is the number of land use categories. *S* is the area of a specific land use type; *i, and j* represent the land use types at two adjacent time points.

### 2.4 Vegetation dynamics

#### 2.4.1 Temporal trend analysis

In studies of vegetation dynamics, regression analysis is widely applied due to its ability to effectively characterize long-term trends (Wang et al., 2013). For a set of observations where time (*x*) serves as the independent variable and the NDVI (*y*) serves as the dependent variable, this study used a linear regression (Equation 2) to describe the overall temporal trend throughout the study period. Scatter plots and fitted trend lines were generated in Origin 2022.

(2) y=a+kx+ϵ

Where *a*, *k* are regression coefficients; *ϵ* indicates the residuals of the linear fitting.

#### 2.4.2 Spatial pattern analysis

Based on the distribution of average *NDVI_ymax_*, and referred to other studies on vegetation cover conditions in the relevant region (Chen Z, et al., 2025), vegetation greenness was classified into five levels: extremely low (<0.1), low [0.1, 0.2), moderate [0.2, 0.4), high [0.4, 0.6), and extremely high (>0.6). Additionally, only grid cells with *NDVI_ymax_* >0.1 were included in the temporal analysis to avoid uncertainty from non-vegetated surfaces (Wei et al., 2025).

### 2.5 Estimation of ESs in IRB

Ecosystem services represent the benefits ultimately generated by ecological processes and functions, which require long-term ecological succession and biogeochemical cycling before they can manifest as substantial and stable changes in service provision. Meanwhile, the riparian forests of the Irtysh River Basin are natural, old-growth forests that have persisted for more than a century. To match the temporal scale of ecosystem processes, this study selected three time points spanning 1990–2020 (1990, 2005, and 2020) and evaluated four key ecosystem services. Four key ecosystem services representing water retention, soil conservation, habitat quality, and carbon storage were quantified using the InVEST model.

The InVEST model requires several empirical parameters, including biophysical coefficients, carbon density, habitat suitability, threat weights, and sensitivity scores. To maximize regional applicability, parameter values were preferentially obtained from field observations and published studies conducted within the Irtysh River Basin and adjacent arid regions of northwestern China. Where regional measurements were unavailable, default values recommended by the InVEST User Guide and validated by previous studies conducted in comparable dryland ecosystems were adopted. Because model outputs may vary with parameter settings, the results should be interpreted as relative spatial patterns rather than absolute ecosystem service quantities.

#### 2.5.1 Water retention

The annual water yield was estimated using the InVEST Water Yield (WY) model based on the Budyko water–energy balance framework (Lyu et al., 2020), which integrates parameters such as annual precipitation, annual potential evapotranspiration, maximum root depth, plant-available water content, land cover type, and biophysical coefficients (**Supplemantary Appendix Table A1**). The core equation (Equations 3–7) are as follows::

(3) $$Y_{xj}=\left(1-\frac{AET_{xj}}{P_{x}}\right)\times P_{x}$$

Where *Y_xj_* is the water yield of land use type *j* and pixel *x* (mm); *AET_xj_* is the actual evapotranspiration (mm) of land use type *j* and pixel *x*; *P_x_* is the precipitation (mm) of pixel *x*.

(4) $$\frac{AET_{xj}}{P_{x}}=\frac{1+\omega_{x}R_{xj}}{1+\omega_{x}R_{xj}+1/R_{xj}}$$

Where *R_xj_* are the Budyko index of dryness for land use type *j* and pixel *x* (Zhang et al., 2004), dimensionless, represents the ratio between annual potential evapotranspiration $ET_{o_{x}}$ (mm) and precipitation *P_x_* (mm); *ω_x_* is the dimensionless ratio of plant available water *AWC_x_* content to accumulated annual precipitation *P_x_* (dimensionless).

(5) $$R_{xj}=\frac{k_{xj}\times ET_{o}x}{P_{x}}\;,\;k_{xj}=\text{min}\left(1,\frac{LAI}{3}\right)$$

Where *k_xj_* is the evaporation factor for land use type *j* and pixel *x*; $ET_{o_{x}}$ is the reference evapotranspiration of pixel *x* (mm).

(6) $$\omega_{x}=Z\times \frac{AWC_{x}}{P_{x}}$$

Where *AWC_x_* is the volumetric plant available water content (mm); *Z* is the seasonality factor, based on model testing experience and relevant research findings (Fu et al., 2017), when the Zhang coefficient is equal to 9, the deviation for simulated and observed water yield is the smallest.

(7) $$AWC_{x}=min\left(max\;Soil\;Depth_{x},\;Root\;Depth_{x}\right)\times PWAC_{x}$$

Where *max Soil Depth_x_* is maximum soil depth (mm); *Root Depth_x_* is soil depth (mm); PAWC is the plant available water capacity (0–1).

Considering that the typical distribution of riparian forests is located in the piedmont region of the Altai Mountains, it is necessary to incorporate the effects of topographic factors (such as elevation and slope) on water retention. Therefore, the annual water yield was corrected using the flow velocity coefficient, topographic index, and saturated soil hydraulic conductivity. The final water retention (WR) was obtained as follows (Equations 8–10):

(8) $$WY_{xj}=\min\left(1,\frac{249}{\text{Velocity}}\right)\times \min\left(1,\frac{0.9\times TI}{3}\right)\times \min\left(1,\frac{K_{sat}}{300}\right)\times Y_{xj}$$

Where *WY_xj_* is water retention amount (mm); *Velocity* is the flow velocity coefficient (**Supplemantary Appendix Table A2**); *TI* is the topographic index (dimensionless); *K*sat is the saturated hydraulic conductivity of soil (cm·d^−1^) (Li et al., 2009); *Y_xj_* is the annual water yield (mm).

(9) $$TI=lg\;\left(\frac{Drainage\_Area}{Soil\_Depth\times Percent\_Slope}\right)$$

Where *Drainage_Area* is the number of contributing grid cells (dimensionless); *Soil_Depth* is the soil depth (mm); *Percent_Slope* is the percentage slope.

(10) In(Ksat)=20.62−0.96×In(Clay)−0.66×In(Sand)−0.46×In(OC)−8.43×BD

Where *Clay* is the clay content of the soil (%); *Sand* is the sand content of the soil (%); *OC* is the soil organic carbon content (%); *BD* is the bulk density of the soil (g·cm^−3^).

#### 2.5.2 Soil conservation

The InVEST Sediment Delivery Ratio (SDR) model was used to soil erosion at each pixel, soil retention by downstream vegetation, and sediment export (Pérez-Cutillas et al., 2026). The core equation (Equations 11–13) are as follows:

(11) $$SDR_{i}=\frac{SDR_{max}}{1+exp\left(\frac{IC_{0}-IC_{i\;\;}}{k}\right)}$$

Where *SDR_i_* is the sediment delivery ratio of the pixel *i*; *SDR_max_* is the maximum SDR (%), and *k* are the calibration parameters; *IC_i_* is the connectivity index of pixel *i*.

(12) $$SE_{i}=RUSLE_{i}\times SDR_{i}$$

(13) $$SE=\sum_{i}SE_{i}$$

Where *SE_i_* is the sediment export (t·ha^−1^·yr^−1^) from pixel *i*; *SE* is the total catchment sediment export (t·ha^−1^·yr^−1^).

Potential soil loss was calculated using the Revised Universal Soil Loss Equation (RUSLE), then adjusted for vegetation cover and soil conservation management to compute actual soil loss. The difference between the two represents the soil retention amount, as expressed in the (Equations 14–20):

(14) $$A=R_{i}*K_{i}*LS_{i}*C_{i}*P_{i}$$

(15) $$\Delta A=R_{i}*K_{i}*LS_{i}$$

(16) $$SR_{i}=\Delta A-A$$

Where *A* is the actual annual soil loss (t·ha^−1^·yr^−1^); *R_i_* is the rainfall erosivity factor (MJ·mm·ha^−1^·h^−1^·yr^−1^); *K_i_* is the soil erodibility factor (t·ha·h·ha^−1^·MJ^−1^·mm^−1^); *LS_i_* is the slope and steepness factor; *C_i_* is the crop management factor; *P_i_* is the support practice factor (**Supplemantary Appendix Table A3**); *LS_i_*, *C_i_*, and *P_i_* are both unitless; Δ*A* is the potential annual soil loss (t·ha^−1^·yr^−1^); *SR_i_* is the amount of annual soil retention (t·ha^−1^·yr^−1^).

(17) $$R=\sum_{i=1}^{12}\left\{1.735\times 10^{\left(1.5\mathrm{lg}\frac{p_{i}2}{p}-0.8188\right)}\right\}$$

Where *p* is annual precipitation (mm); *p_i_* is monthly precipitation (mm).

(18) $$K=0.1317\times \left(-0.01383+0.51575\times K_{epic}\right)$$

(19) $$K_{epic}=\left\{0.2+0.3\exp\left[-0.0256SAN\left(1-\frac{SIL}{100}\right)\right]\right\}\times {\left(\frac{SIL}{CLA+SIL}\right)}^{0.3}\times \left[1-\frac{0.25C}{C+\exp\left(3.72-2.95\text{C}\right)}\right]\times \left[1-\frac{0.7SNI}{SNI+\exp\left(22.9SNI-5.51\right)}\right]$$

(20) $$SNI=1-\frac{SAN}{100}$$

Where *SAN*, *SIL*, and *CLA* are the sand, silt, and clay fractions (%); *C* is the soil organic carbon content (%).

#### 2.5.3 Habitat quality

The Habitat Quality (HQ) model in InVEST estimates the spatial extent and degradation level of habitats and vegetation based on the distribution of land cover types, the intensity of habitat threats, and the sensitivity of habitats to stressors (Moreira et al., 2018). The habitat quality index ranges from 0 to 1. The calculation The core equation (Equation 21, 22) are as follows:

(21) $$Q_{xj}=H_{j}\left(1-\frac{D_{xj}^{z}}{D_{xj}^{z}+K^{z}}\right)$$

Where *Q_xj_* is habitat quality score of land use type *j* in pixel *x*; *H_j_* is habitat suitability level of land use type j (dimensionless, 0-1); *Z* and *K* are scaling constants, *Z* is typically set to 2.5, and *K* is half-saturation constant, with a default value of 0.5.

(22) $$D_{xj}=\sum_{r=1}^{R}\sum_{y=1}^{Y_{r}}\left(\frac{\omega_{r}}{\sum_{r=1}^{R}\omega_{r}}\right)r_{y}i_{rxy}\beta_{x}S_{jr}$$

Where *D_xj_* is habitat degradation of land use type *j* in pixel *x*; *R* is the total number of threat factors (**Supplementary Appendix Table A4**); *Y_r_* is the total number of pixels corresponding to threat factor *r*; *ω_r_* is the weight of threat factor *r*; *r_y_* is intensity of threat factor *r* in pixel y (0 or 1); *i_rxy_* represents the distance decay of threat factor *r* from pixel *y* to pixel *x*; calculated using either linear decay (Equation 23) or exponential decay (Equation 24). *β_x_* is the accessibility level of threat factor *r* to pixel *x* [0, 1]; *S_jr_* is the sensitivity score of land use type *j* to threat factor *r* (**Supplementary Appendix Table A5**), ranging from 0 to 1.

(23) $$i_{rxy}=1-\left(\frac{d_{xy}}{d_{r,max}}\right)$$

(24) $$i_{rxy}=\text{exp}\left(-2.99d_{xy}/d_{r,max}\right)$$

Where *d_xy_* is the distance between pixel *y* and pixel *x*; (m); *d_rmax_* is the maximum influence distance of threat factor *r*.

#### 2.5.4 Carbon storage

The Carbon Storage and Sequestration (CS) model in InVEST depends on four carbon pools: aboveground biomass, belowground biomass, soil organic carbon, and litter carbon (Liu et al., 2021a). For each land cover type, the carbon density parameters of the four pools are assigned, and the total regional carbon storage is obtained as (Equations 25, 26):

(25) $$C_{m}=C_{m\_above}+C_{m\_below}+C_{m\_soil}+C_{m\_dead}$$

(26) $$C_{total}=\sum_{m=1}^{n}C_{m}\times S_{m}$$

Where *C_m_* is total carbon density in land use type *m* (t·hm^−2^) (**Supplementary Appendix Table A6**); *C_m_above_* is aboveground biomass carbon in land use type *m* (t·hm^−2^); *C_m_below_* is belowground biomass carbon in land use type *m* (t·hm^−2^); *C_m_soil_* is soil organic carbon in land use type *m* (t·hm^−2^); *C_m_dead_* is dead organic matter carbon in land use type *m* (t·hm^−2^); *C_total_* represents the sum of carbon storage across four carbon pools within each land use type; *n* is the total number of land use types; *S_m_* indicates the area of the *m_th_* land use type.

#### 2.5.5 Overall benefit of ecosystem services

Since different ESs are interrelated, an integrated assessment is required to evaluate their overall benefits. To avoid uncertainties associated with subjective weighting and to emphasize the equal fundamental importance of each service within the integrated ecosystem service framework, an equal-weighting method was applied. In the absence of clearly defined ecological priorities, the overall benefit of ecosystem services was calculated by equally weighting the four standardized services (Qian et al., 2023). The data standardization method is as follows:

Min-Max Normalization is applied to eliminate dimensional differences among indicators by scaling them to a common range [0, 1]. This preprocessing enables comparison among indices with different units or magnitudes. Data normalization was performed in ArcGIS 10.8 using the raster calculator, as shown in (Equation 27):

(27) $$y_{ij}=\frac{X_{ij}-minX_{j}}{maxX_{j}-minX_{j}}$$

where *y_ij_* is the standardized value of the *jth* indicator in the ith sample; *xij* (*i* = 1, 2, …, n; *j* = 1, 2, …, m) is the *jth* indicator in the *ith* sample, *maxX_j_* and *minX_j_* are the maximum and minimum of the *jth* indicator, respectively.

The comprehensive ecosystem service was calculated as (Equation 28):

(28) $$ES_{total}=\frac{1}{n}\sum_{i-1}^{n}{ES}^{\prime }$$

where *ES_total_* is the combined benefits of *n* ESs and $ES_{i}^{\prime }$ is the standardized value of the *ith* ES.

### 2.6 Statistical analysis and model validation

Path Analysis combines quantitative correlations with qualitative causal relationships to elucidate how variables interact and influence one another (Gong et al., 2024; Hollar, 2018). To clarify the ecological pathways linking environmental conditions, land use and cover transformation, vegetation condition, and ecosystem service provision, observed-variable path analysis was employed. This method directly estimates hypothesized causal relationships among observed ecological variables, and is particularly suitable when all variables are directly measurable indicators.

#### 2.6.1 Variable selection

The natural environmental conditions and human activities jointly regulate the provision of ecosystem services (Bai et al., 2022). To characterize the ecological pathways of hydrologically constrained riparian forest ecosystems in arid and semi-arid regions, this study selected six representative variables, including land use and cover, vegetation condition, and other driving factors. Among them, the other driving factors selected four indicators: (1) total annual gross domestic product (GDP); (2) elevation (DEM); (3) mean annual precipitation (PRE); and (4) mean annual potential evapotranspiration (PET) were selected to represent socio-economic scale, terrain constraints, water input, and atmospheric water demand, respectively. It should be noted that land use and cover is represented by land use intensity (abbreviated as LUCC) in this context. Based on previous Literature (Zhang et al., 2025), the intensity level of construction land, arable land, and all other land use types were assigned values of 3, 2, and 1, respectively. Vegetation condition (abbreviated as NDVI) was measured by annual maximum NDVI. ES represented overall benefit of ecosystem services.

#### 2.6.2 Pathway specification

The conceptual pathway model was established *a priori* based on ecological theory and previous empirical studies rather than solely according to statistical relationships (**Table 2**). Specifically, topography (DEM), precipitation (PRE), and potential evapotranspiration (PET) were regarded as the primary natural environmental drivers controlling landscape dynamics and vegetation condition. Gross domestic product (GDP) was included as an indicator of human activities influencing land use transition. Land use transition was assumed to directly regulate vegetation condition by altering habitat conditions, while vegetation condition was hypothesized to mediate the influence of land use and cover transformation on ecosystem service provision. Integrated ecosystem service provision was therefore considered the final ecological response variable in the pathway model.

**Table 2:** Hypothesized ecological relationships among variables.

Variable	Ecological role	Expected effect
Topography (DEM)	Terrain constraint	±
Precipitation (PRE)	Water availability	+
Potential evapotranspiration (PET)	Atmospheric water demand	−
Gross domestic product (GDP)	Human disturbance	±
Land use transition (LUCC)	Landscape transformation	±
Vegetation condition (NDVI)	Ecological mediator	+
Integrated ecosystem service (ES)	Ecological response	/

#### 2.6.3 Model evaluation

Prior to conducting the path analysis, the 3 km × 3 km fishnet grid (326 cells) was generated in ArcGIS 10.8 (**Supplementary Appendix Figure B2**), and the values of all variables were extracted at grid centroids. All variables were standardized to eliminate dimensional differences. Pearson correlation analysis was conducted to examine pairwise relationships among variables, and all explanatory variables satisfied commonly accepted thresholds (variance inflation factors< 5), indicating that multicollinearity was unlikely to affect parameter estimation. Standardized path coefficients were estimated using multiple regression analyses implemented in SPSS 19.0, and statistical significance was determined at P< 0.05.

## 3 Results

### 3.1 Spatiotemporal differentiation characteristics of long-term land use and cover patterns

#### 3.1.1 Spatial distribution patterns and changes in land use and cover

**Figure 2:**
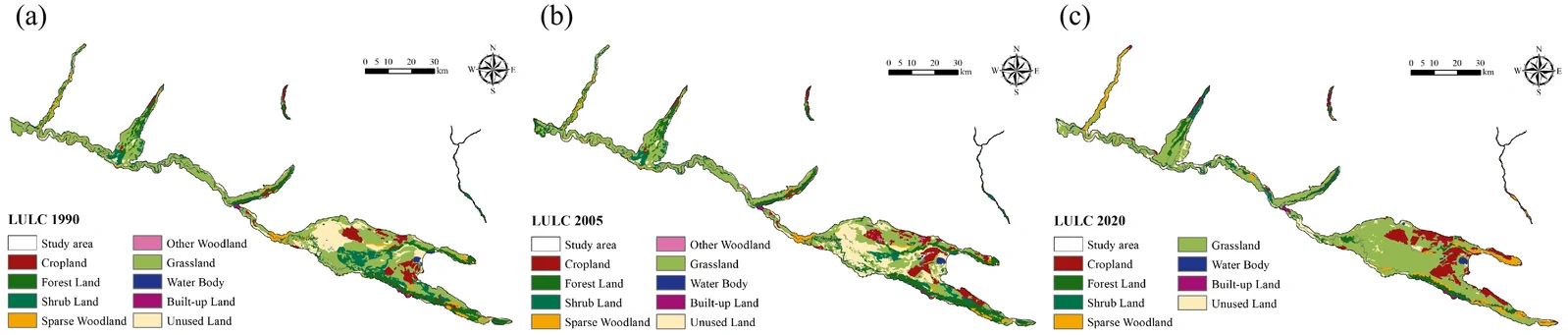
Land use and cover types in the riparian forest of the IRB during **(a)** 1990, **(b)** 2005, and **(c)** 2020.

Grassland, cropland, and forest land were the predominant LULC types in the riparian forest in the valley area of the IRB (**Figure 2**). Cropland was mainly concentrated around the 181th TuanChang. Forest land was continuously distributed along both sides of the river channels, particularly in the Beitun reach of the main stream Irtysh River and the middle reaches of the Burqin and Haba rivers. Compared with 1990 and 2005, unused land, which was originally clustered at the confluence of the Crane River and the Irtysh River, gradually became fragmented and concentrated in the lower reaches of the Crane River. Other woodland was the only land use type that disappeared completely by 2020.

As shown in **Table 3**, grassland accounted for 48.30% of the riparian forest area in 1990, followed by unused land and forest land (14.36% and 13.99%, respectively). Shrub land, cropland, sparse woodland, and water bodies accounted for 8.53%, 6.56%, 4.07%, and 3.51%, respectively, whereas other woodland and built-up land constituted less than 1%. From 1990 to 2020, grassland remained the dominant land use type, increasing to 61.90% of the total area by 2020. At the same time, cropland expanded to 12.74%, whereas forest land, shrub land, and unused land decreased significantly, falling to 6.40%, 1.56%, and 5.81%, respectively.

**Table 3:** The areas (km^2^) and proportions (%) of different land use and cover types in the riparian forest area of the IRB during 1990–2020.

Year	Statistical parameter	Land use and cover types				
		Cropland	Forest land	Shrub land	Sparse woodland	Other woodland
1990	Area	153.96	328.30	200.16	95.60	0.11
	Proportion	6.56	13.99	8.53	4.07	0.01
2005	Area	196.84	382.10	122.86	79.95	5.37
	Proportion	8.39	16.28	5.23	3.41	0.23
2020	Area	299.08	150.31	36.55	148.43	0
	Proportion	12.74	6.40	1.56	6.32	0.00
Year	Statistical Parameter		Land use and cover types			
			Grassland	Water body	Built-up land	Unused land
1990	Area		1133.88	82.35	15.79	337.19
	Proportion		48.30	3.51	0.67	14.36
2005	Area		996.07	90.43	16.46	457.18
	Proportion		42.44	3.85	0.70	19.48
2020	Area		1453.20	107.20	16.24	136.25
	Proportion		61.90	4.57	0.69	5.81

**Figure 3:**
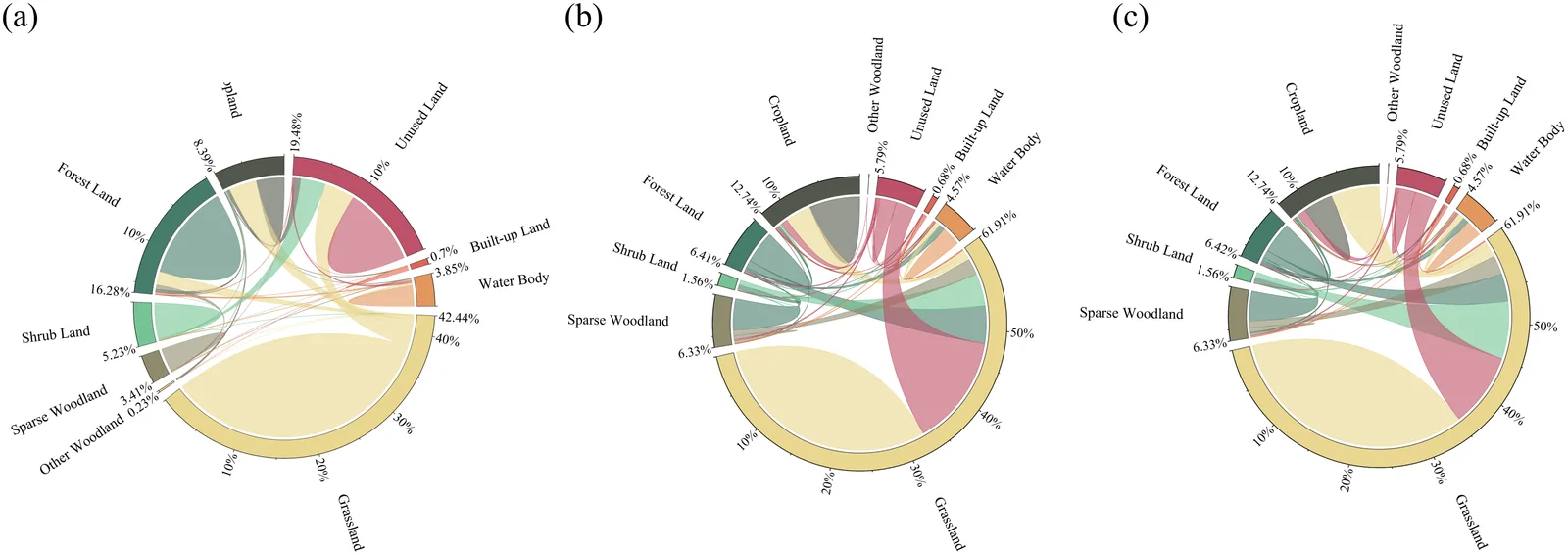
Land use and cover transitions in the riparian forests of the IRB **(a)** 1990–2005, **(b)** 2005–2020, and **(c)** 1990–2020.

Land use transitions (**Figure 3**) revealed a frequent and complex pattern of mutual conversion among all LULC types between 1990 and 2020. Forest land was predominantly transformed into sparse woodland and grassland, and only 27.76% of forest land remained unchanged during this period. Substantial shifts in the land use and cover structure have occurred across the entire 30-year period, and the overall transition characteristics from 2005 to 2020 (**Figure 3b**) was similar to those from 1990 to 2020 (**Figure 3c**). The consistency suggests that the major conversion pathways established after 2005 persisted over the long-term period.

#### 3.1.2 Transitions and changes of woodland classification types

Long-term land use transition substantially reshaped the landscape composition of riparian forests throughout the Irtysh River Basin during the study period. **Table 4** summarizes the subtypes of woodland and their areas across different riparian forest zones. Except for the Berezek River, all river regions experienced a decline in total woodland area over the 30-year period, especially after 2005. The decline was most pronounced along the main stream Irtysh River. By 2020, the total woodland areas ranked as follows: Irtysh River > Crane River > Haba River > Berezek River > Burqin River. forest land was most extensively distributed along the Irtysh River (79.36 km^2^), whereas no forest land was recorded in the Berezek River region.

**Table 4:** Woodland classification types and areas in different riparian forest zones of the IRB at different periods (km^2^).

Riparian forest zone	Year	Woodland				Total area
		Forest land	Shrub land	Sparse woodland	Other woodland	
Irtysh River	1990	151.34	140.67	60.70	0	352.71
	2005	192.16	68.95	48.87	0	309.98
	2020	79.36	16.27	67.47	0	163.10
Burqin River	1990	44.30	0	5.93	0	50.23
	2005	44.27	0	5.95	0	50.22
	2020	22.90	1.62	0	1.44	25.96
Crane River	1990	65.55	26.39	14.02	0.03	105.99
	2005	77.69	20.89	10.11	5.35	114.04
	2020	13.10	0	48.78	0	61.88
Haba River	1990	48.36	32.89	2.86	0	84.11
	2005	48.46	32.87	2.89	0	84.22
	2020	34.76	18.50	0	0	53.26
Berezek River	1990	18.22	0	11.93	0	30.16
	2005	18.81	0	11.98	0	30.79
	2020	0	0.10	30.62	0	30.72

**Figure 4:**
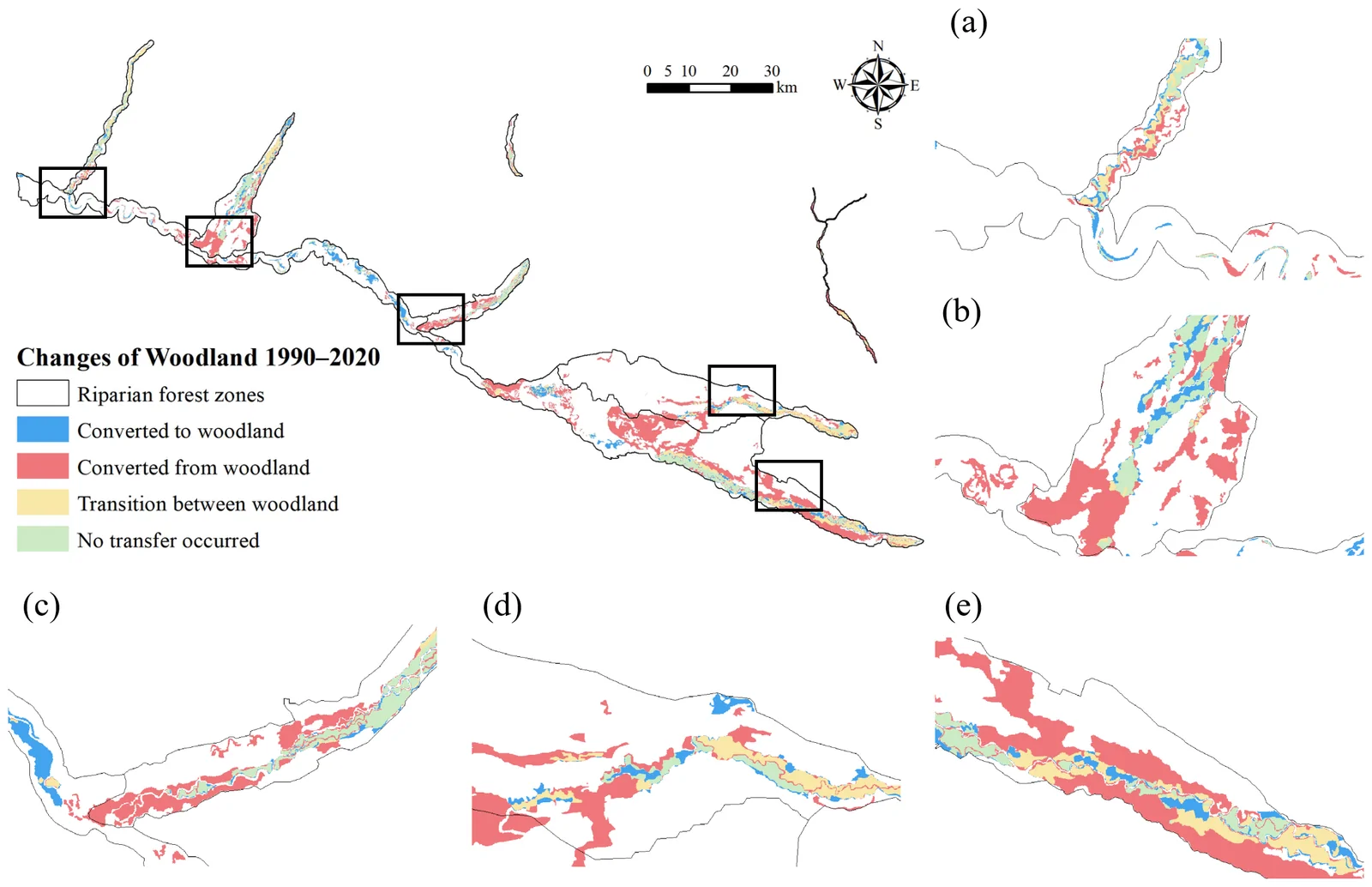
Spatiotemporal patterns of woodland expansion and loss in different riparian forest zones of the IRB during 1990–2020 **(a)** Berezek River, **(b)** Haba River, **(c)** Burqin River, **(d)** Crane River, and **(e)** Irtysh River. The orientation of the north arrow in the enlarged sub-figures is consistent with that in the original figure, and the scale is 1/10 of the original.

The spatial distribution of woodland expansion and loss is presented in **Figure 4**. (1) Berezek River: dominated by transitions among woodland subtypes, and woodland loss primarily occurred in downstream reaches. (2) Haba River: woodland expansion occurred in the upper reaches. Extensive woodland remained in the middle reaches, while significant loss concentrated around Qibal Town in the downstream section. (3) Burqin River: woodland loss and unchanged areas were dominant, and large contiguous patches of loss extended from the middle reaches to the confluence with the Irtysh River. (4) Crane River: mainly characterized by transitions among woodland subcategories, with changes concentrated in the Khesebaihar and 181th TuanChang sections in the middle reaches. (5) Irtysh River: all four types of woodland transitions occurred. Woodland expansion originated near the confluence with the Burqin River and extended along the northern bank toward the confluence with the Haba River. Woodland loss clustered in large patches around the confluence with the Crane River and near the Jiaosate Village. The Beitun reach remained largely unchanged.

### 3.2 Spatiotemporal differentiation characteristics of vegetation structure dynamics

#### 3.2.1 Spatial distribution patterns and changes in vegetation greenness

**Figure 5:**
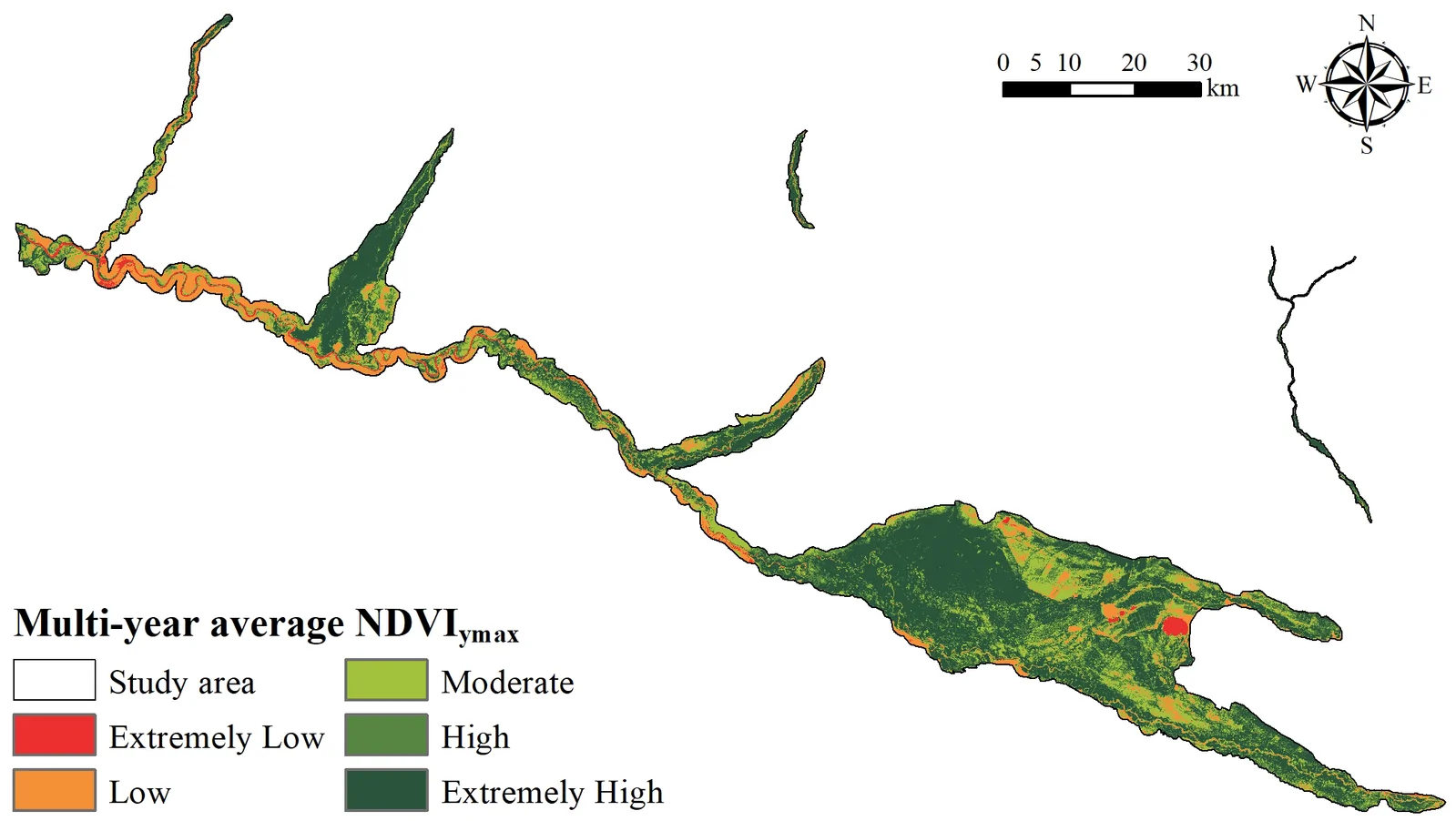
Spatial distribution of multi-year average *NDVI_ymax_* in the riparian forest in the valley area of the IRB.

Vegetation condition exhibited pronounced spatial and temporal responses to long-term landscape transformation. The multi-year average *NDVI_ymax_* exhibited stable spatial patterns in extremely high and extremely low categories, whereas changes in other categories primarily occurred around the 181th TuanChang (**Figure 5**). Extremely high vegetation greenness showed prominent linear distribution along both sides of the main stream Irtysh River (Xibo Ferry–Akebulegen Village), the Crane River (Kizgar Reservoir–181 th TuanChang), and the Buerjin River.

As summarized in **Table 5**, the average multi-year *NDVI_ymax_* was 0.50, indicating relatively high vegetation greenness. In terms of area, vegetation greenness categories ranked: extremely high > moderate > high > low > extremely low. Extremely high and extremely low greenness covered 1,057.65 km^2^ and 81.54 km^2^, respectively.

**Table 5:** The area (km^2^) and proportions (%) of different vegetation greenness grades and multi-year average *NDVI_ymax_* in the riparian forest in the valley area of the IRB.

Statistical parameter	Vegetation greenness grades					Multi-year average *NDVI_ymax_*		
	Extremely low	Low	Moderate	High	Extremely high	Max	Min	Average
Area	81.54	338.51	443.63	424.33	1057.65			
Proportion	3.48	14.43	18.91	18.09	45.09			
						0.90	-0.19	0.50

#### 3.2.2 Temporal variation and trend of the vegetation index (*NDVI_ymax_*)

**Figure 6:**
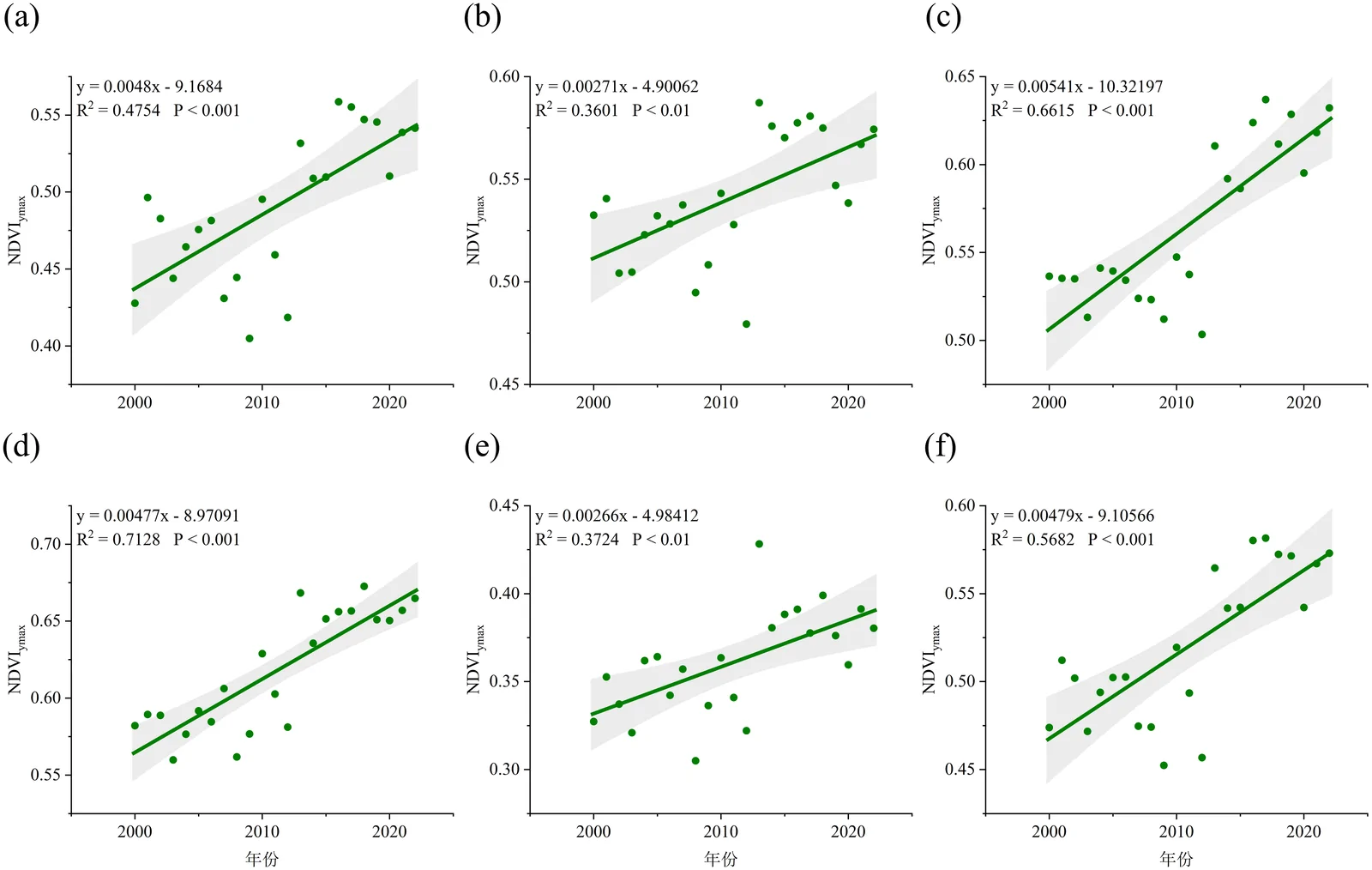
Interannual variation and linear trends of *NDVI_ymax_* (2000–2022) in different riparian forest zones of the IRB **(a)** the mainstream Irtysh River, the tributaries **(b)** Burqin River, **(c)** Crane River, **(d)** Haba River, **(e)** Berezek River, and **(f)** all rivers. Green dots represent the annual average NDVIymax, the green lines indicate the fitted trend line, and the gray area represents the 95% confidence interval.

The interannual variation of *NDVI_ymax_* is shown in **Figure 6**. Across all riparian forest zones, *NDVI_ymax_* ranged from 0.45 to 0.58, reaching its lowest value in 2009 and peaking in 2017, with a significant increasing trend over time (R^2^ = 0.5682, P< 0.001). The minimum *NDVI_ymax_* was observed in the Berezek River (0.31, in 2008), whereas the maximum occurred along the Haba River (0.67, in 2018). All riparian forest zones exhibited increasing trends, with the Haba River showing the strongest linear relationship (R^2^ = 0.7128, P< 0.001).

### 3.3 Spatiotemporal differentiation characteristics of ecosystem services

**Figure 7:**
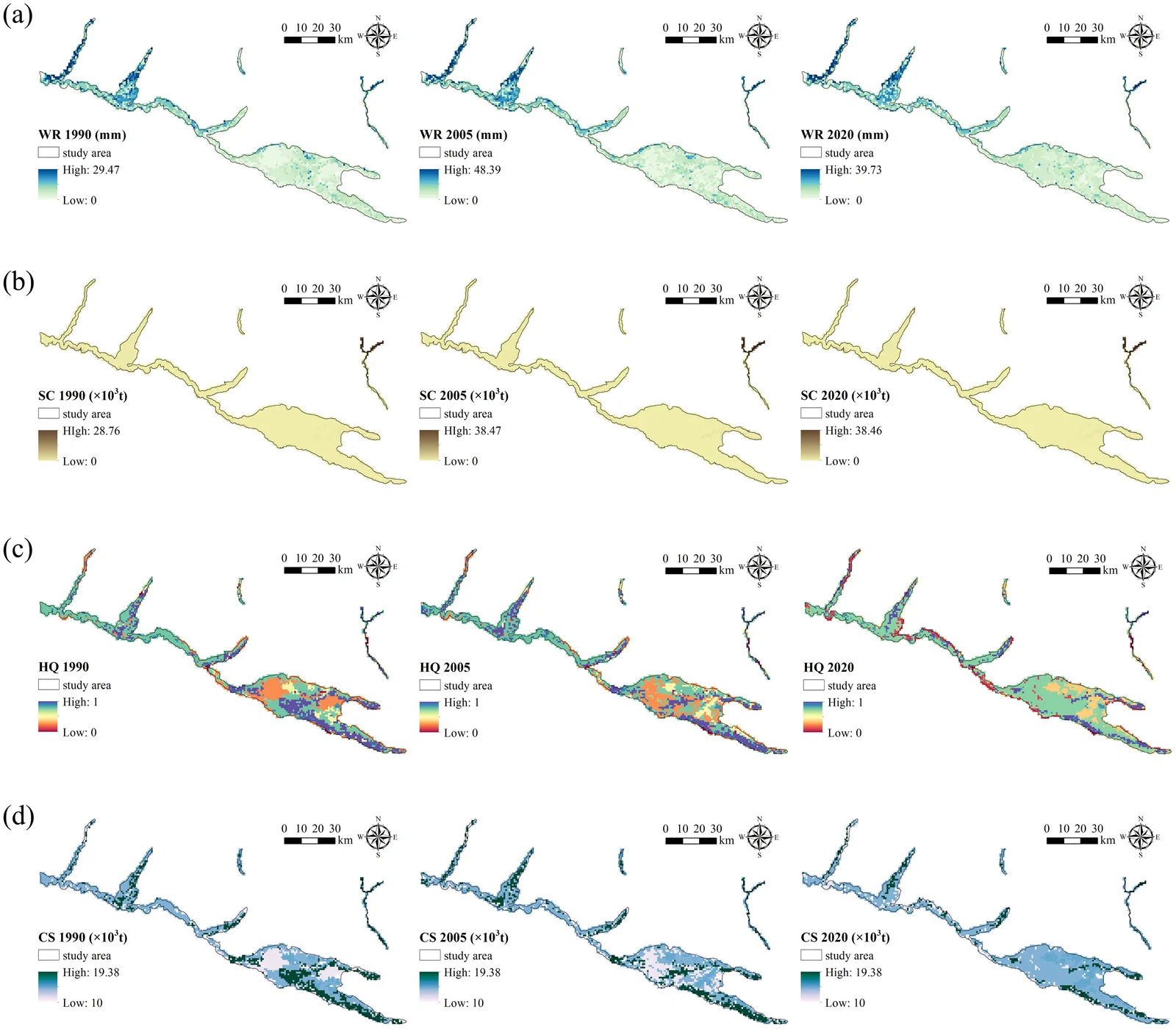
Spatial distribution of ecosystem services in riparian forest of the IRB during 1990–2020 **(a)** WR represents water retention, **(b)** SC represents soil conservation, **(c)** HQ represents habitat quality, and **(d)** CS represents carbon storage. .

Four ecosystem services exhibited pronounced spatial heterogeneity across the riparian forest in the valley area of the IRB (**Figure 7**), with wide variations in value ranges (**Table 6**). Water retention, habitat quality, and carbon storage displayed highly uneven spatial distributions, while soil conservation showed weaker heterogeneity. High soil conservation values were concentrated in the upper Crane River, while low values dominated the plains.

**Table 6:** The average per unit area and total values of ecosystem services in the riparian forests of the IRB.

Types of ecosystem services		1990	2005	2020
Water retention	Average per unit area (mm)	4.24	5.47	4.56
	Total value (10^3^ m^3^)	12.60	16.24	13.55
Soil conservation	Average per unit area (t)	168.22	217.24	206.95
	Total value (10^3^ t)	499.78	645.42	614.848
Habitat quality	Average [0-1]	0.71	0.70	0.72
Carbon storage	Average per unit area (t)	9515.76	9400.52	9143.60
	Total value (10^5^ t)	282.71	278.29	271.66

High-value areas of water retention were primarily distributed along the Berezek River channel, the middle reaches of the Haba River, and the upper Crane River. Low-value areas clustered between the lower Crane River and the upper Irtysh River, gradually diminishing over time. Habitat quality and carbon storage showed similar patterns: high-value areas were located on the western banks of the Haba and Burqin rivers, the middle–upper Crane River, and the Xibo Ferry–Beitun section of the mainstream Irtysh River. Low-value areas were concentrated near Kalatale and Baluwangtas villages, where cropland and intensive human activity dominated. By 2020, low-value habitat quality areas expanded markedly, especially along the confluence sections of the Irtysh, Crane, and Haba rivers, and in the middle–upper Berezek River region.

Over the 30-year period, water retention and habitat quality remained relatively stable. By 2020, the average unit area WR in the study area was 4.56 mm, while HQ remained high at 0.72. SC displayed a trend of increasing followed by decreasing, and the average unit area SC peaking at 217.24×10^3^ t in 2005 before declining to 206.95×10^3^ t in 2020. The average unit area CS showed a continuous decline from 9515.76×10^5^ t (1990) to 9143.60×10^5^ t (2020). Collectively, these results indicate that ecosystem services responded heterogeneously but generally exhibited consistent long-term improvement.

### 3.4 Ecological pathways of ecosystem services and their influencing factors

**Figure 8:**
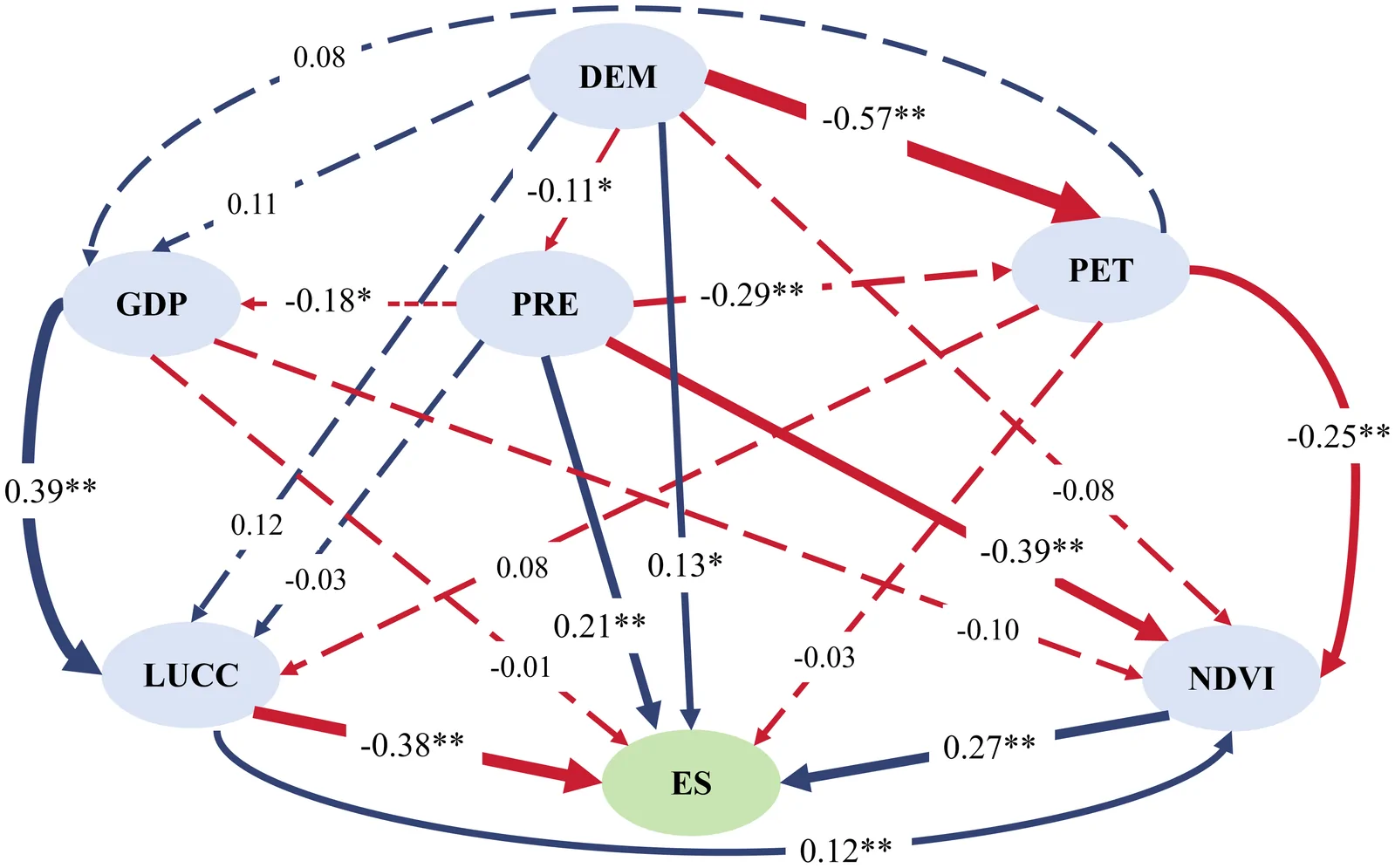
The path analysis for the effects of different factors on ecosystem service. Blue and red arrows represent positive and negative effects, respectively. Line width of arrows reflecting the magnitude of the path coefficients. Solid lines indicate significant, while dashed lines indicate non-significant. The number indicates the path coefficients (direct effects), represent the strength and direction of the relations between variables. * and ** are indicate statistical significance at P< 0.05 and P< 0.01, respectively.

Path analysis (**Figure 8**) demonstrated that land use intensity (LUCC) exerted a significant negative effect on integrated ecosystem services with the path coefficient -0.38, whereas vegetation greenness (NDVI) showed a significant positive effect with the path coefficient 0.27. DEM and PRE also exhibited significant positive influences (path coefficients were 0.13 and 0.21, respectively). GDP and PET had no significant direct effects on ecosystem services. Additionally, GDP significantly promoted LUCC, which in turn positively influenced NDVI. DEM was negatively correlated with both PRE and PET. PRE and PET significantly negatively affected NDVI. These patterns indicate that integrated ecosystem service provision reflects the cumulative ecological consequences of land use and convey transformation and vegetation condition.

## 4 Discussion

### 4.1 Changes in forest extent are more sensitive along the main stream of the Irtysh River

The expansion and loss of forest land are driven jointly by natural processes and human activities, and their distribution is often spatially heterogeneous along river courses. Forest expansion typically occurs in headwater regions, upper river reaches, areas subject to periodic flooding (Keram et al., 2019), and zones near protected reservoirs or water-conservation areas (Yin et al., 2017). Over the past 30 years, notable transitions in forest land have occurred across the riparian forest regions of the IRB. Significant forest loss was detected in the desert areas near Salhusun Township, as well as at the confluences of the Irtysh, Burqin, and Haba Rivers. At the same time, forest expansion was also concentrated mainly along the main stream of the Irtysh River.

The opposing trends: forest expansion in the middle reaches and forest loss in the upper–middle reaches, reflecting the spatial imbalance of anthropogenic impacts along the main stream. Two major drivers explain this pattern: firstly, within the basin system, the main stream plays a dominant geomorphological and hydrological role. Compared with tributaries, the main stream typically features a wider and flatter valley floor, larger catchment area, and longer flood peaks, which together enhance both scouring processes and opportunities for vegetation establishment. Secondly, the main stream is characterized by higher population density and more accessible transportation corridors. During land use transitions and policy shifts, such as the implementation of natural forest protection and ecological red-line delineation, these areas experienced stronger drivers of forest change. As a result, areas that had previously suffered forest loss have shown signs of restorative expansion. The coexistence of these contrasting trends highlights the complexity of basin-scale ecological processes and the spatial–temporal heterogeneity of human influence.

### 4.2 Declines in arid and semi-arid forest area do not necessarily imply decreases in vegetation greenness

The GIMMS–NDVI 3g dataset, with its global coverage and long-term consistency, has been widely applied in studies of vegetation dynamics (Goetz et al., 2006; Li et al., 2017, 2023). However, its coarse spatial resolution (1/12°) limits its ability to capture fine-scale variations within the riparian forests of the IRB. Although medium-resolution MODIS NDVI data have been used to characterize spatiotemporal NDVI patterns and drivers in the Altay region between 2000 and 2013 (Wei et al., 2015), research specifically focused on riparian forest vegetation dynamics remains scarce.

Our results indicate that the *NDVI_ymax_* in all riparian forest in the valley area of the IRB remained at a relatively high level, fluctuating between 0.45 and 0.58. Differences exist among river with the lowest value (0.31) observed along the Berezek River and the highest (0.67) along the Haba River. In terms of temporal variation characteristics, *NDVI_ymax_* exhibited an overall increasing trend from 2000 to 2022, with a pronounced minimum in 2009. During the growing season (April–August), precipitation in riparian vegetation zones is only about 87 mm. Consequently, vegetation growth relies more on the irrigation effects of overbank flooding to meet its water demand (Deng, 2023). From the perspective of actual channel runoff conditions in the Irtysh River Basin, problems such as frequent channel siltation and flow interruption have been reported (Nuerlan·Jialieli, 2017). However, as the Irtysh River is a transboundary river, its historical hydrological data are not fully publicly available. By comparison, the long-term trend in water level changes of Ulungur Lake, located within the same geographical region, also showed the lowest recorded water level in 2009. Therefore, the specific hydrological event that occurred in 2009 was considered to be one of the key factors contributing to the simultaneous occurrence of the lowest *NDVI_ymax_* value in that year.

In dryland riparian forests, vegetation greenness may be decoupled from forest area due to compensatory vegetation dynamics. By integrating vegetation dynamics with land use and cover change analysis, we find that reductions in forest area are not equivalent to reductions in vegetation greenness. The fundamental reason is that NDVI reflects vegetation greenness, same as biomass and physiological vigor, rather than vegetation cover per se (Cao et al., 2024). Thus, qualitative improvements in vegetation can offset or even surpass quantitative losses in forest area. On the one hand, while total forest extent declined, remaining trees received more light, water, and nutrients, improving their growth conditions. Additionally, reduced grazing pressure, restricted construction activities, and ecological restoration initiatives further enhanced vegetation vitality. On the other hand, forested areas converted into cropland or grassland were frequently replaced by high-biomass crops or vigorous pioneer herbaceous species, elevating *NDVI_ymax_* across the broader region. This finding suggests that NDVI alone may overestimate vegetation recovery in riparian ecosystems where land use and cover composition changes substantially.

### 4.3 Ecosystem services are jointly driven by land use intensity and vegetation greenness

Previous studies have identified mountain zones in the Altay region as hotspots of ecosystem service importance (Fu et al., 2016). Our findings show strong spatial heterogeneity in water retention, habitat quality, and carbon storage within the riparian forests in the valley area of the IRB, with carbon storage reaching high levels. High-value carbon storage was distributed mainly along the Haba and Burqin Rivers, the upper–middle reaches of the Crane River, and along the Irtysh River between Xibo Ferry and Beitun. From 1990 to 2020, water retention and habitat quality remained relatively stable, whereas soil conservation and carbon storage showed varying degrees of decline. Notably, soil retention increased markedly in 2005, forming a distinct peak primarily driven by adjustments in land use policies. Soil retention services are highly sensitive to vegetation cover and the soil-stabilizing capacity of root systems. Around 2000, large-scale ecological restoration programs implemented in northwestern China, such as the Grain-for-Green Program and the Natural Forest Protection Program, promoted localized recovery or enclosure-based restoration of riparian forests, resulting in significant short-term improvements. However, after 2005, with the expansion of agricultural development and increasing constraints on water resources, vegetation stability declined and the risk of soil erosion increased, leading to a subsequent reduction in soil retention services. These findings indicate that ecosystem services of riparian forests in arid regions exhibit pronounced stage-dependent responses and high sensitivity to human disturbances.

Our findings support the ecosystem service cascade hypothesis. Beyond documenting land use and cover changes, this study advances the ecosystem service cascade perspective by explicitly linking land use transition, vegetation dynamics, and ecosystem service provision within a unified ecological pathway framework. Studies within the same geographical area indicated that ecosystem services in the Altay region have been negatively impacted by land use change (Fu et al., 2017). The declining trend in habitat quality was strongly associated with land use intensity (Lu et al., 2024). While in arid and semi-arid climates, NDVI exerts a positive influence on integrated ecosystem services (Qian et al., 2023). In the riparian forests of the IRB, our results similarly reveal significant negative and positive effects of land use intensity and vegetation greenness, respectively, on integrated ecosystem services. These observations also emphasize that ecosystem services originate from ecosystem structures through ecosystem functioning rather than from land use and cover alone, and vegetation condition (NDVI) partially mediates this relationship.

It is worth noting that elevation shows a negative correlation with both annual average precipitation and annual average potential evapotranspiration. Annual average precipitation and annual average potential evapotranspiration, in turn, show significant negative effects on NDVI. The influence of elevation on precipitation and evapotranspiration is governed by large-scale geographical and climatic controls. The Irtysh River Basin is an ecologically fragile zone in northwestern China (Liu et al., 2022), and the riparian forests are mainly located on the piedmont plains of the Altay Mountains. Low elevations characterized by warmer and drier conditions compared with mountainous terrain. Consequently, precipitation and evapotranspiration decrease with altitude. NDVI negative associations with precipitation and evapotranspiration may appear paradoxical. However, riparian forests predominantly grow in floodplain zones where water availability is tightly controlled by snowmelt processes (Zhang et al., 2017). Surface runoff is largely derived from the meltwater of winter snowpack. During early forest growth stages, precipitation often falls as snow and is not readily available to vegetation. As temperatures rise, snowmelt and evapotranspiration increase, but human activities (e.g., agricultural irrigation) restrict the availability of ecological water for riparian forests. Therefore, the mismatch between vegetation phenology and hydrological rhythm results in simultaneous high water demand and water scarcity. This phenomenon constitutes a core feature of riparian forest ecosystems in the IRB.

### 4.4 Hierarchical governance strategies for optimizing riparian forest ecosystem services in the Irtysh River Basin

Over the past decades, major ecological restoration programs such as the Three-North Shelterbelt Program, and the Grain for Green Program have achieved significant progress in ecological governance within the IRB. Nevertheless, ecological problems such as localized riparian forest degradation and simplified stand structures still persist. Meanwhile, adaptive management measures including floodplain irrigation techniques and enclosure-based forest protection have also produced certain positive outcomes. However, the combined effectiveness of these multiple measures has not yet been fully realized. Therefore, it is necessary to adopt a system-oriented approach, moving beyond single-objective management models and exploring a general methodological framework to address challenges in riparian forest protection and restoration decision (**Table 7**). Specifically:

**Table 7:** Principles and scientific basis of multi-category governance strategies.

Category	Governance principles	Scientific basis
Structure	Priority to pattern stability	Forest expansion is scattered and difficult to scale
	Prevent structural simplification	Forest conversion mainly occurs toward grassland and cropland
Function	Hydrology-vegetation coordinated restoration	Vegetation dynamics differ significantly among tributaries
	Risk-prevention orientation	NDVI levels remain relatively low in some areas
Service	Priority to long-term stability of multiple services	Different ecosystem services exhibit complex change trends
	Improve integrated management mechanisms	Integrated ecosystem services are jointly driven by natural and social factors

#### 4.4.1 Structural category of the ecosystem: implementing adjustment-oriented regulation strategies to optimize ecological spatial

1. Existing forest land and continuously distributed forest patches along both sides of rivers should be regarded as the structural foundation of riparian forest ecosystems. Particular attention should be given to river sections where forest patches still maintain a certain degree of connectivity, including the Beitun section of the Irtysh River, the upper and middle reaches of the Burqin River, and the upper and middle reaches of the Haba River.
2. Strict control should be imposed to prevent grassland and cropland from encroaching into the core zones of floodplain forests, especially in regions where forest land has historically been frequently converted into grassland or cropland (e.g., the lower reaches of the Haba River), structural adjustment measures oriented toward grazing exclusion and cropland-to-forest restoration should be explored to gradually restore the appropriate spatial proportion and hierarchical structure of riparian forests.

#### 4.4.2 Functional category of the ecosystem: restoration-oriented regulation to stimulate ecosystem vitality and enhance self-maintenance and regeneration capacity

1. Riparian forests possess a certain degree of natural resilience. Given that riparian forest growth and development are strongly influenced by floodplain water availability, it is essential to ensure adequate ecological inundation flows and sufficient water release duration to meet the water requirements of trees during critical growth stages in the downstream sections of the Irtysh River. Enhancing natural restoration capacity under such hydrological conditions is a key factor for improving NDVI levels.
2. Particular attention should be paid to the Berezek River, where NDVI levels remain relatively low. Priority should be given to addressing problems such as weakened tree growth and slow regeneration of young trees. On the one hand, stand competition dynamics should be clarified to optimize and adjust forest stand structures. On the other hand, artificial supplementary planting should be implemented to promote the natural regeneration process of riparian forests.

#### 4.4.3 Service category level: construction-oriented regulation to actively guide and strengthen specific ecosystem services

1. Habitat quality currently represents the dominant ecosystem service of riparian forests. It is therefore important to avoid conflicts among ecosystem services caused by excessive multi-objective stacking. In river sections such as the Xibo Ferry-Beitun reach of the Irtysh River, the middle reaches of the Crane River, the upper reaches of the Burqin River, and the middle reaches of the Haba River, efforts should focus on consolidating their ecological functions as biodiversity conservation areas and species migration corridors.
2. Under conditions of uneven distribution of integrated ecosystem service supply within the basin, management should emphasize following ecological processes and reducing excessive engineering interventions, while promoting the establishment of ecological compensation mechanisms between upstream and downstream sections of the same river and among different rivers. Meanwhile, local forestry management departments and stakeholders should be encouraged to jointly develop riparian forest management plans, enhance public participation and awareness of ecosystem service values, and integrate local advantages to develop value-added activities such as ecological volunteer conservation and ecotourism.

In summary, the hierarchical governance strategy of ecosystem services proposed in this study follows a progressive pathway of “pattern stabilization- functional enhancement-service synergy.” Our suggestions emphasize that effective riparian management should prioritize not only forest area conservation but also vegetation quality improvement and ecological water allocation. Although this study focused on the Irtysh River Basin, the proposed analytical framework is applicable to many arid and semi-arid riparian ecosystems experiencing simultaneous ecological restoration and sustainable watershed management.

### 4.5 Limitations and future prospects

#### 4.5.1 Data and scale limitations

This study was conducted at the watershed scale using multi-source remote sensing datasets. While this scale is appropriate for revealing long-term regional ecological dynamics, it cannot fully capture fine-scale ecological processes, groundwater–vegetation interactions, species-level responses, or seasonal ecosystem dynamics. In addition, this study only clarified the land use and cover patterns at several representative time points, which may have overlooked the ecological impacts of some short-term human disturbances or natural fluctuations.

#### 4.5.2 Parameter uncertainty in ecosystem service assessment

Ecosystem service assessment was conducted using the InVEST model, whose parameterization inevitably involves uncertainties associated with regional transferability. Although model parameters were derived from widely adopted datasets and published studies applicable to arid and semi-arid ecosystems, local field calibration was not available for all ecosystem service modules. Therefore, inevitably contains uncertainties associated with regional parameter transferability.

#### 4.5.3 Methodological limitations of ecological pathway analysis

The ecological pathways identified in this study were quantified using observed-variable path analysis. Although this approach effectively evaluates hypothesized direct and indirect relationships between driving factors and ecosystem service provision. However, ecosystem degradation and restoration are inherently dynamic processes characterized by cumulative effects, threshold responses, and time-lag effects. Consequently, linear path analysis may underestimate nonlinear ecological responses driven by hydrological disturbances or ecological restoration policies. Therefore, the proposed ecological pathways should be interpreted as theory-driven ecological relationships.

#### 4.5.4 Future prospects

Understanding how landscape transformation regulates ecosystem services remains one of the central challenges in ecosystem service research. Owing to the inherent complexity of social–ecological systems, no single study can fully characterize the multiple processes governing ecosystem service dynamics. At present, riparian forests in the valley area of the Irtysh River Basin have shifted from the initial realization of ecological restoration to precise decision-making for sustainable management under complex systems. Future research should place greater emphasis on integrating ecological monitoring, process-based analysis, and adaptive management frameworks.

Several research priorities deserve particular attention: (1) integrating long-term field observations, high-resolution remote sensing, and regional parameter optimization would further improve the accuracy of ecosystem service estimation; (2) future studies should strengthen the dynamic simulation of riparian forests ecological processes and scenario-based predictions, such as evaluating ecological risks, restoration potential, thereby providing scientific support for optimization of medium and long-term zoning control boundaries; (3) increasing attention should be paid to cross-boundary ecosystem service flows and ecological compensation mechanisms, which are critical for coordinating conservation and development across administrative boundaries. Such efforts will enhance the resilience and sustainability of riparian forests under the combined pressures of global environmental change and human activities.

## 5 Conclusion

Using land use transition matrices, univariate linear regression, the InVEST model, and path analysis, this study elucidates the spatial–temporal patterns of water retention, soil conservation, habitat quality, and carbon storage in the riparian forests in the valley area of the Irtysh River Basin from 1990 to 2020, and clarifies the complex interactions among integrated ecosystem services, land use intensity, vegetation greenness, and other drivers. The main conclusions are:

1. Thirty years of land use transition reshaped the riparian forest land use and cover structure, characterized by grassland expansion, cropland increase, and forest loss, yet grassland remains dominant, accounting for 61.9% of the study area. In 2020, forest area ranked as follows: Irtysh River > Crane River > Haba River > Berezeke River > Burqin River. The main stream of the Irtysh exhibited the largest forest extent (79.36 km^2^) and the highest sensitivity to both expansion and loss.
2. Despite reductions in forest area, vegetation greenness generally improved, suggesting a decoupling between vegetation structure and vegetation functioning. Long-term *NDVI_ymax_* across all forest zones remained high (0.45–0.58). The overall greenness of vegetation across individual forest zones showed an upward trend, with the lowest value (0.31) recorded along the Berezeke River in 2008. The spatial distribution patterns of extremely high and extremely low vegetation greenness remained stable, while other categories changed mainly in the 181st TuanChang area.
3. Multiple ecosystem services exhibited heterogeneous spatial responses. Except for soil conservation, the other three ecosystem services showed clear spatial heterogeneity, with the high-value areas were concentrated along the Berezeke, Haba, and upper–middle Crane rivers. Over the 30-year period, water retention and habitat quality remained relatively stable, whereas soil retention and carbon storage declined. In 2020, carbon storage (9143.60×10^5^ t) remained at a relatively high level.
4. Integrated land use and cover transformation, vegetation condition, and ecosystem service provision within a unified ecological pathway framework. Ecosystem services were significantly affected by land use intensity (negative effect) and vegetation greenness (positive effect), with path coefficients of –0.38 and 0.27, respectively. Topography and hydrothermal conditions also indirectly influenced ecosystem services by modulating vegetation growth.

By tracing historical trajectories of land use and cover change, and vegetation dynamics, and by quantifying natural and anthropogenic drivers of integrated ecosystem services, this study deepens the understanding of the ecological functions and value of riparian forests. The findings provide essential insights for policymakers and interested parties seeking to design effective strategies for riparian forest conservation and restoration.

## Data Availability

The original contributions presented in the study are included in the article/**Supplementary Material**. Further inquiries can be directed to the corresponding author.
