# Supplementary material for: Spatiotemporal evolution and ecological pathways of riparian forests within the ecosystem service cascade framework: a comprehensive analysis from the Irtysh River Basin, China
Source: Front Plant Sci. 2026 Aug 19;17:1904769. doi: 10.3389/fpls.2026.1904769 (PMC13534071; doi:10.3389/fpls.2026.1904769)
Supplement: Supplementary file 1 [file DataSheet1.docx]

Supplementary Material

# 1 Appendix A

| **TABLE A1** Biophysical parameter table of water yield module. | | | | | | | | | | | | | | | | | | |  |
| --- | --- | --- | --- | --- | --- | --- | --- | --- | --- | --- | --- | --- | --- | --- | --- | --- | --- | --- | --- |
| Lucode | | LULC_desc | | | | LULC_veg | | | | Root_depth | | | | Kc | | | | |  |
| 1 | | Cropland | | | | 1 | | | | 2100 | | | | 0.5 | | | | |  |
| 2 | | Woodland | | | | 1 | | | | 5200 | | | | 0.8 | | | | |  |
| 3 | | Grassland | | | | 1 | | | | 2000 | | | | 0.5 | | | | |  |
| 4 | | Water Body | | | | 0 | | | | 100 | | | | 1 | | | | |  |
| 5 | | Built-up Land | | | | 0 | | | | 100 | | | | 0.1 | | | | |  |
| 6 | | Unused Land | | | | 0 | | | | 300 | | | | 0.1 | | | | |  |
| **TABLE A2** Flow velocity coefficients used in the water retention model. | | | | | | | | | | | | | | | | | | | |
| *Vel_coef* | | | Land use/Land cover Types | | | | | | | | | | | | | | | | |
|  |  |  | Cropland | Woodland | | | | Grassland | | | Water Body | | | | Built-up Land | | Unused Land | | |
|  | | | 800 | 180 | | | | 400 | | | 2012 | | | | 2012 | | 1500 | | |
| **TABLE A3** *C* and *P* factors used in the soil conservation model. | | | | | | | | | | | | | | | |  |  |  |  |
| *Lucode* | *LULC_desc* | | | | | | *ulse_c* | | | | | *ulse_p* | | | |  |  |  |  |
| 1 | Cropland | | | | | | 0.05 | | | | | 0.3 | | | |  |  |  |  |
| 2 | Woodland | | | | | | 0.03 | | | | | 1 | | | |  |  |  |  |
| 3 | Grassland | | | | | | 0.04 | | | | | 1 | | | |  |  |  |  |
| 4 | Water Body | | | | | | 0 | | | | | 0 | | | |  |  |  |  |
| 5 | Built-up Land | | | | | | 0 | | | | | 0 | | | |  |  |  |  |
| 6 | Unused Land | | | | | | 1 | | | | | 1 | | | |  |  |  |  |
| **TABLE A4** Threat factors of habitat quality and their attributes. | | | | | | | | | | | | | | | | | |  |  |
| Threat source type | | | | | Weight | | | | Max. influencing distance(km) | | | | Spatial decay   type | | | | |  |  |
| Cropland | | | | | 0.6 | | | | 6 | | | | linear | | | | |  |  |
| Built-up Land | | | | | 0.8 | | | | 8 | | | | exponential | | | | |  |  |
| Unused Land | | | | | 0.7 | | | | 5 | | | | linear | | | | |  |  |

| **TABLE A5** Habitat suitability of different land use types and sensitivity to threat sources. | | | | | | | | | |
| --- | --- | --- | --- | --- | --- | --- | --- | --- | --- |
| Lucode | LULC_desc | Habitat suitability | | Threat source | | | | | |
|  |  |  |  | Cropland | | Build-up land | | Unused Land | |
| 1 | Cropland | 0.5 | | 0 | | 0.7 | | 0.4 | |
| 2 | Woodland | 1 | | 0.7 | | 0.85 | | 0.5 | |
| 3 | Grassland | 0.8 | | 0.7 | | 0.8 | | 0.7 | |
| 4 | Water Body | 0.9 | | 0.45 | | 0.7 | | 0.4 | |
| 5 | Built-up Land | 0 | | 0 | | 0 | | 0 | |
| 6 | Unused Land | 0.3 | | 0.4 | | 0.55 | | 0 | |
| **TABLE A6** Carbon density parameters used in the carbon storage model (t/hm^2^). | | | | | | | | |  |
| Lucode | LULC_desc | | C_above | C_below | C_soil | | C_dead | |  |
| 1 | Cropland | | 3.3 | 9.7 | 85.1 | | 0.8 | |  |
| 2 | Woodland | | 9.3 | 7.1 | 176.1 | | 1.3 | |  |
| 3 | Grassland | | 5.7 | 13.7 | 67.9 | | 0.5 | |  |
| 4 | Water Body | | 0.1 | 0 | 0 | | 0 | |  |
| 5 | Built-up Land | | 0.3 | 0 | 0 | | 0 | |  |
| 6 | Unused Land | | 0.2 | 0 | 19.9 | | 0 | |  |

# 2 Appendix B

The delineation of typical riparian forest distribution areas was based on buffer zones established along the river centerlines: main stream (1) Irtysh River: 1200 m; major tributary (2) Burqin River: 1000 m; tributary (3) Crane, (4) Haba, and (5) Berezek River: 600 m. Additionally, upper Burqin River and upper Crane River were 500 m and100 m, respectively. These boundaries were refined based on previous studies (Section 2.1.2).

**FIGURE 1**

Delineation of the study area

A fishnet grid refers to a feature class composed of regular rectangular cells. The create fishnet tool in ArcGIS was used to divide the study area into uniform analytical units for spatial analysis and data management.

**FIGURE 2**

3 km×3 km fishnet grid of the study area
